# Metabolic Profile Associated With Encystation in *Acanthamoeba*

**DOI:** 10.1111/jeu.70034

**Published:** 2025-07-30

**Authors:** Cecília Cirelli, Isabela Aurora Rodrigues, Jéssica Gardone Vitório, Filipe Fideles Duarte‐Andrade, Gisele André Baptista Canuto, Leiliane Coelho André, Juliano Simões de Toledo, Ana Paula Fernandes, Adriana Oliveira Costa

**Affiliations:** ^1^ Departamento de Análises Clínicas e Toxicológicas, Faculdade de Farmácia Universidade Federal de Minas Gerais Belo Horizonte Brazil; ^2^ Departamento de Clínica, Patologia e Cirurgia Odontológicas, Faculdade de Odontologia Universidade Federal de Minas Gerais Belo Horizonte Brazil; ^3^ Departamento de Química Analítica, Instituto de Química Universidade Federal da Bahia Salvador Brazil

**Keywords:** a*canthamoeba*, encystation, gas chromatography–mass spectrometry, metabolomics

## Abstract

The genus *Acanthamoeba* includes widespread protozoa that can cause severe infections in humans. Their ability to form resistant cysts within infected tissues complicates treatment, making it essential to understand the encystation process for developing effective therapeutic strategies. This study utilized untargeted metabolomics (GC–MS) to analyze metabolic changes during the encystation of an *Acanthamoeba* strain in Neff's encystation saline. We conducted metabolite analysis at three stages of differentiation: the trophozoite‐dominant phase (0 h), the pre‐cyst‐dominant phase (24 h), and the cyst‐dominant phase (72 h). The results indicated a global metabolic downregulation during encystation, which is consistent with a state of dormancy. Components of the cyst wall such as cellobiose and lactose accumulated in the final phase. Arbutin and canavanine were annotated for the first time in *Acanthamoeba*. Encystation also led to changes in pathways related to glycine, serine, and threonine metabolism and biosynthesis of aminoacyl‐tRNA. This study uncovered previously unknown metabolites and metabolic pathways at distinct stages of *Acanthamoeba* development.

## 1 Introduction

*Acanthamoeba* spp. are free‐living protozoa ubiquitously found in aquatic and terrestrial environments, such as soil, air samples, air conditioning systems, dust, and hospital equipment (Marciano‐Cabral and Cabral 2003). These species are opportunistic pathogens that can cause severe human infections, such as amoebic keratitis (AK) and granulomatous amoebic encephalitis (GAE). AK is predominantly related to wearing contact lenses, having a prolonged, tiring treatment, and posing a risk of partial or total vision loss (Büchele et al. 2023). In GAE, the active stages invade the central nervous system and determine a condition of high mortality, generally in immunocompromised individuals (Khan 2006; Kalra et al. 2020). The relative rarity of *Acanthamoeba* infections, if compared to those caused by other pathogens, results in late or mistaken diagnosis, worsening the clinical condition and the disease.

The life cycle of *Acanthamoeba* consists of two main stages: trophozoites, the active, feeding stage characterized by spine‐like pseudopodia (*acanthopodia*), and cysts, the dormant, resistant stage with a metabolically inactive state (Marciano‐Cabral and Cabral 2003; Garajová et al. 2019). Encystation is a survival mechanism triggered by environmental stressors, including nutrient deprivation, desiccation, and extreme pH or temperature fluctuations (Marciano‐Cabral and Cabral 2003). This process also occurs during human infections, contributing to drug resistance and disease recurrence (Khan 2006; Aksozek et al. 2002; Lloyd 2014). Cysts also pose a significant challenge in AK prevention, as they can remain viable despite exposure to biocides used in contact lens solutions (Walters et al. 2022).

The cyst wall, composed of a double layer, confers a significant resilience to *Acanthamoeba* (Chávez‐Munguía et al. 2005). During encystation, an amorphous layer—the ectocyst—forms externally, later adopting a fibrillar structure. The inner layer or endocyst develops subsequently with cellular rounding and cytoplasmic dehydration. The mature cyst wall contains specialized pore‐like structures called ostioles. These appear as constricted regions where the ectocyst and endocyst layers converge and form opercula, which serve as the exit points for the trophozoite during excystation (Bowers and Korn 1969; Chávez‐Munguía et al. 2005). Cellulose, the primary structural component, is critical for the cyst's resistance (Garajová et al. 2019; Lee et al. 2015).

Recent advances in omics technologies have revolutionized our capacity to understand genotype–phenotype relationships. Metabolomics, in particular, represents the most downstream component of the omics cascade by examining the terminal products of metabolism, offering a direct link to phenotypic responses (Johnson and Siuzdak 2016; Tounta et al. 2021). While these approaches have transformed many areas of biological research, their application to protist differentiation remains limited. Bernard et al. (2022) pioneered a time‐resolved transcriptomic, proteomic, and phosphoproteomic atlas of *Acanthamoeba* encystation, revealing rapid phosphorylation‐mediated signaling and transcriptional regulation preceding proteomic changes, though their study did not include metabolomic analysis. In *Acanthamoeba* spp., a couple of existing metabolomic studies employed targeted strategies, one focusing on sterol biosynthesis pathways (Zhou et al. 2018) and another analyzing amino acid homeostasis in *Acanthamoeba* under amoeba‐bacteria coculture conditions (Tsai et al. 2023). To address a more comprehensive understanding of the metabolic changes during stage differentiation, we conducted an untargeted metabolomic analysis of an *Acanthamoeba* strain encystation using gas chromatography–mass spectrometry (GC–MS). We analyzed metabolic profiles at three critical time points during encystation induction, providing clear differences in the metabolic profiles of *Acanthamoeba* stages.

## 2 Materials and Methods

### 2.1 *Acanthamoeba* Culture and Encystation

The reference strain ATCC 30010 used in this study was historically identified as *A. castellanii* but recent phylogenetic findings redefined it as *A. terricola* (Corsaro et al. 2024). Trophozoites were cultivated at 32°C in a Peptone‐Yeast‐Glucose (PYG) culture medium, pH 6.5, as previously described (Costa et al. 2021) with regular passages twice a week. Cultures of 72 h in PYG were washed three times using Neff's encystation saline (95 mM NaCl, 100 mM KCl, 8 mM MgSO_4_, 0.4 mM CaCl_2_, 1 mM NaHCO_3_, and 20 mM Tris–HCl, pH 9.0) (Neff et al. 1964) by centrifugation at 400 g at room temperature. Trophozoites (1 × 10^7^) were added to culture flasks containing 10 mL of Neff's saline to induce encystation. Following 24, 48, and 72 h of incubation at 32°C, three replicate flasks were transferred to an ice bath for 10 min. Cells were gently detached using a cell scraper, and developmental stages (trophozoites, pre‐cysts, and cysts) were quantified by manual counting in a hemocytometer under a light microscope. Viability assessment by 0.4% Trypan Blue exclusion confirmed ≥ 92% viability across all stages. The following morphological features were considered to quantify the stages: acanthopodia and contractile vacuoles in trophozoites, rounded shape and single‐layered wall in pre‐cysts, and a double‐layered wall in cysts (Lorenzo‐Morales et al. 2008).

### 2.2 Metabolite Extraction

Intracellular metabolites were extracted from cells transferred to Neff's encystation saline at 0, 24, and 72 h of cultivation, using an independent set of flasks for each time point. Metabolic quenching was performed by subjecting the culture flasks to a rapid decrease in temperature for 20 s in an ethanol/dry ice bath. The cells (10^7^) were washed in cold PBS, and the supernatant was removed by centrifugation (4°C, 400 *g*, 10 min). The pellet was suspended in 1.5 mL of methanol pre‐heated at 56°C and incubated at the same temperature for 15 min. Five hundred microliters of deionized water were added to the tubes, and the samples were subjected to 3 cycles of sonication (20 s, 75 Hz). Next, 500 μL of chloroform were added, and three freeze and thaw cycles were performed. The samples were centrifuged (4°C, 16.000 *g*, 10 min), and 200 μL of the supernatant was distributed to 10 microcentrifuge tubes per group (0, 24, and 72 h). The tube contents were completely dried using a SpeedVac concentrator.

The dried samples were derivatized using a previously published method (Cruz et al. 2019). For quality controls (QC), we prepared a pooled sample containing 20 μL aliquots from each specimen and processed it through methoximation (16 h, room temperature) and silylation (1 h, 70°C). This QC pool was injected five times initially for column conditioning, then after every fifth sample injection, and finally at the end of the sequence. Blank samples (no cells) monitored background. This protocol ensured derivatization reproducibility (QC feature RSD < 15%) and instrument stability (retention time drift < 0.1 min).

### 2.3 Metabolomics Analysis and Data Processing

Untargeted metabolomics analyses were performed using a 7890C gas chromatography system, coupled with a 5975A quadrupole mass detector (Agilent Technologies, Santa Clara, CA, USA), equipped with an automatic injector combiPAL autosampler, as previously described (Cruz et al. 2019).

Data processing was performed using XCMS software (version 1.24.1) on an R platform (version 3.2.2). The matched filter method was used for peak detection using the following parameters: (i) peak width (fwhm) = 6; (ii) signal‐to‐noise ratio (snthresh) = 2; (iii) maximum number of peaks per extracted ion chromatogram (max) = 100. Peak grouping was based on bandwidth correction (bw) = 6 and 3 (first and second grouping, respectively), and the width of the overlapped bands of *m/z* (mzwid) = 0.25. The “fillPeaks” tool was used to remove missing values, and the default “retcor” method was applied with nonlinear alignment and smoothing degree of polynomial regression adjustment (span) = 0.5 to correct the retention time. Metabolite annotation was performed in AMDIS (Automated Mass spectral Deconvolution and Identification System) software using the Fiehn GC/MS RLT Library for the correlation of co‐eluted compounds based on the retention index and retention time. The data set was normalized according to the C18:0 methyl stearate internal standard before performing statistical analysis.

### 2.4 Statistical Analysis

Data were Pareto scaled and log_2_ transformed for multivariate analysis. Principal Component Analysis (PCA) and Partial Least Squares Discriminant Analysis (PLS‐DA) were conducted in SIMCA P+ (version 14.1, Umetrics, CA, USA). Univariate pairwise comparisons were performed on Statistica software (version 10), using either the Student *t* test or the Mann Whitney *U* test, according to the normality distribution determined by Lilliefors statistical tests. The threshold for significance was set to a *p* < 0.05. Metabolic pathway analyses were performed in MBrole 2.0, using data deposited in KEGG for the organisms *Dictyostelium discoideum, Entamoeba dispar*, and *Entamoeba histolytica* .

## 3 Results

### 3.1 *Acanthamoeba* Encystation Curve

**FIGURE 1:**
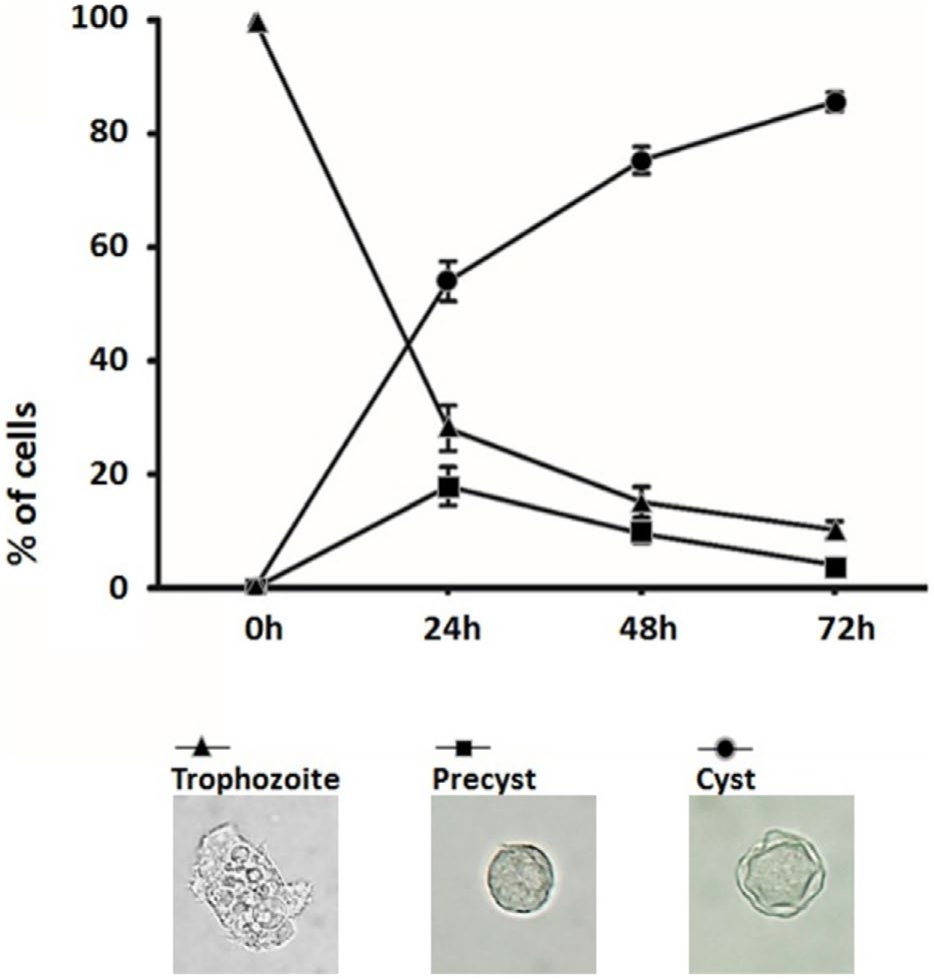
Encystation of *Acanthamoeba* (ATCC 30010) in Neff's encystation saline. Trophozoites (10^7^) were added to culture flasks containing 10 mL of the encystation medium. Quantification of the morphological stages was performed every 24 h in independent sets of flasks (*n* = 3). The data represent the mean and standard error of counts performed in triplicate.

The number of *Acanthamoeba* trophozoites decreased drastically after 24 h in the encystation medium, reaching only 28.1% of the total cells (Figure 1). Meanwhile, the proportions of pre‐cysts and cysts increased to 17.9% and 54%, respectively. By 48 h, trophozoites accounted for just 15.1% of the cells, while pre‐cysts and cysts represented 9.6% and 75.3%, respectively. Finally, at 72 h, cysts were the predominant stage, constituting 85.6% of the total population, whereas trophozoites and pre‐cysts made up only 10.3% and 4.1%, respectively.

### 3.2 Metabolomics Analysis

In a principal component analysis (PCA), the clustering of quality controls confirmed the stability and reproducibility of the analytical technique (Figure S1A). Additionally, the PCA revealed a clear clustering trend of samples based on culture time (Figure S1B). A total of 1205 molecular features (corresponding to mass spectra fragments) were detected, with 23 metabolites successfully annotated (Table S1). These metabolites were primarily classified as amino acids, peptides, and conjugates (47.83%), followed by carbohydrates (26.09%), fatty acids and conjugates (13.04%), organic nitrogen compounds (4.35%), phenols and derivatives (4.35%), and alcohols and polyols (4.35%).

### 3.3 Metabolic Differences Between Trophozoites and Cysts

**FIGURE 2:**
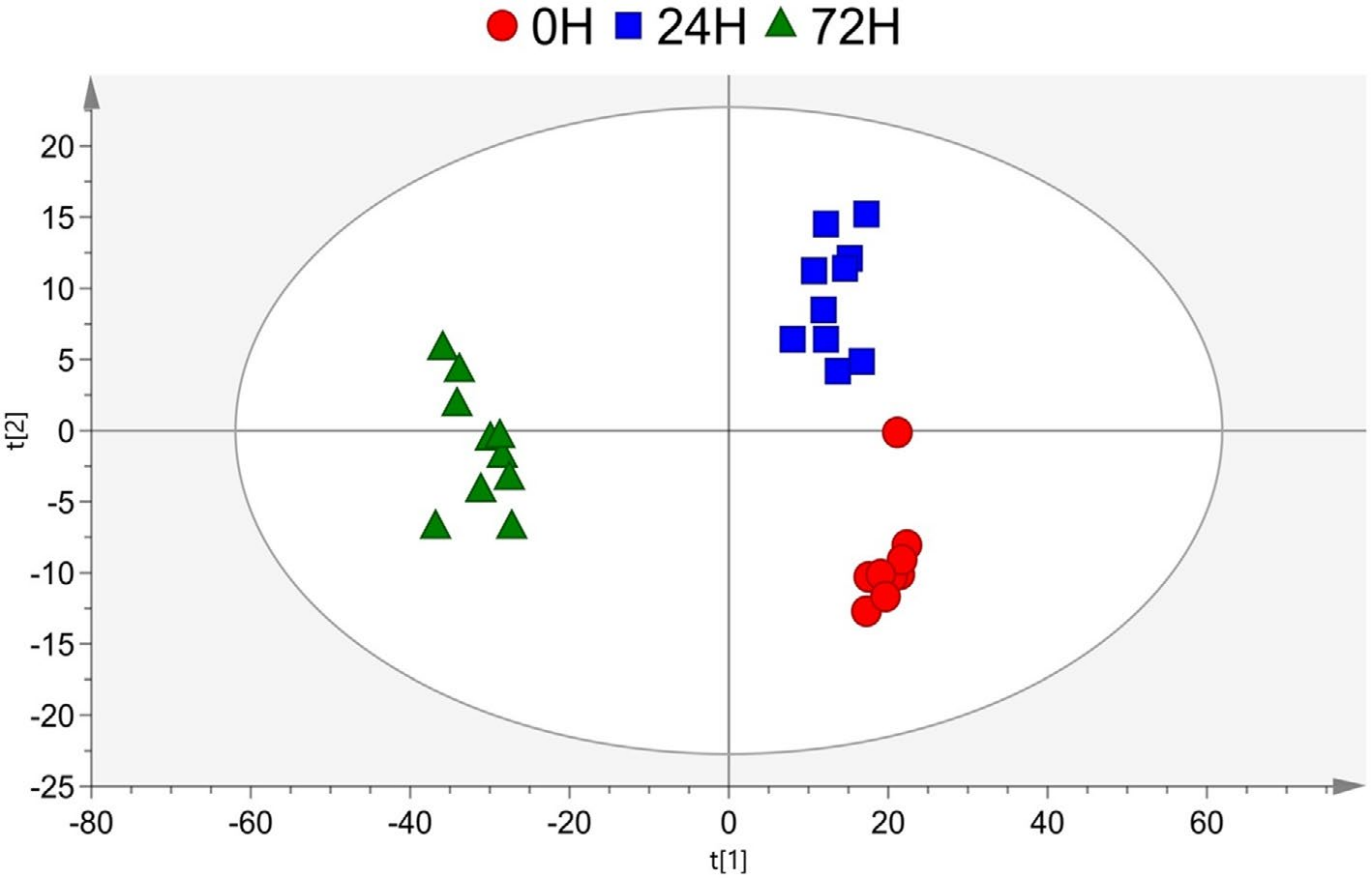
PLS‐DA score plots of identified metabolites from *Acanthamoeba* (ATCC 30010) in Neff's encystation saline at times 0, 24, and 72 h. The PLS‐DA model demonstrated a clustering tendency of samples at distinct times of encystation. Quality parameters of the model: *R* ^2^ = 0.896 and *Q* ^2^ = 0.785, CV‐ANOVA *p*‐value = 4 × 17^−10^.

The metabolomic data collected during trophozoite‐to‐cyst differentiation were analyzed using multivariate methods. A PLS‐DA model was applied to identify discriminative metabolites by comparing different culture times. After 72 h of cultivation in Neff's encystation saline, samples containing predominantly cysts formed a distinct cluster, separating them from cells collected at earlier time points. The model demonstrated a good fit and strong predictive ability (*Q* ^2^ = 0.785, Figure 2), and its statistical significance was confirmed by CV‐ANOVA (*p* = 4.17 × 10^−10^).

**FIGURE 3:**
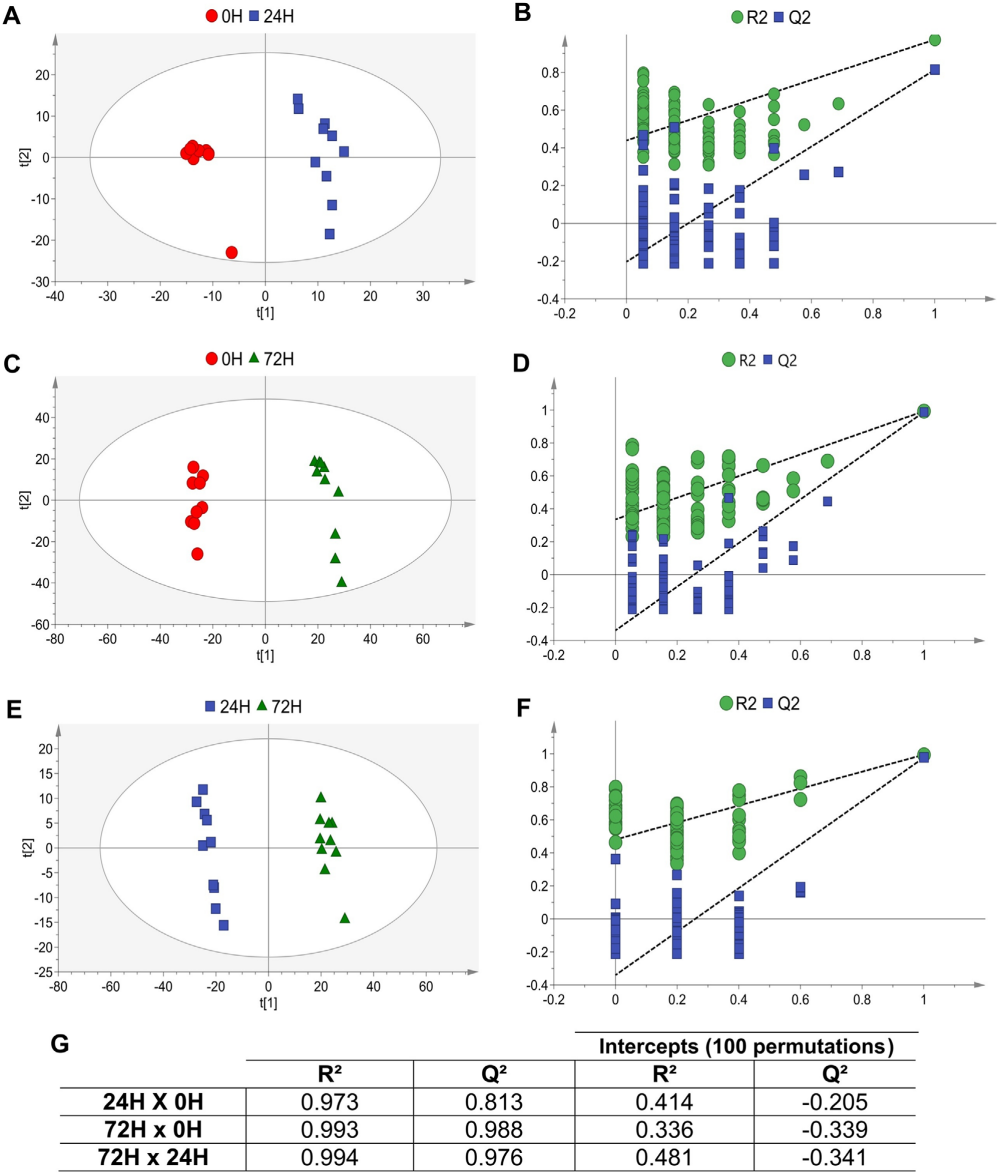
PLS‐DA models comparing the distinct times of encystation of *Acanthamoeba* (ATCC 30010) in Neff's saline in pairs. The models were built with the total features normalized by the intensity of the internal standard C18:0 L, transformed in log with base 2, and pareto scaled. A, C, E: Score plots comparing 0 × 24 h, 0 × 72 h, and 24 × 72 h, respectively. B, D, F: Quality parameters *R* ^2^ (goodness of fit) and *Q* ^2^ (predictive ability) values for 0 × 24 h, 0 × 72 h, and 24 × 72 h models, respectively. G: Table summarizing the exact *R* ^2^, *Q* ^2^, and CV‐ANOVA *p*‐values for all pairwise comparisons. In all score plots (A, C, E), a clear separation between groups is observed, suggesting distinct metabolic profiles at each time point. The high *R* ^2^ and *Q* ^2^ values (B, D, F), along with significant CV‐ANOVA results (G), confirm the robustness and predictive validity of the models.

To further assess metabolic differences across encystation time points, pairwise PLS‐DA models were applied (Figure 3). All models were statistically significant (CV‐ANOVA, *p* < 0.05) and showed clear separation between time points (Figure 3A–C), consistent with the trends observed in Figure 2. High model quality was further supported by *R* ^2^ > 0.9 and *Q* ^2^ > 0.8 (Figure 3B,D,F,G).

**FIGURE 4:**
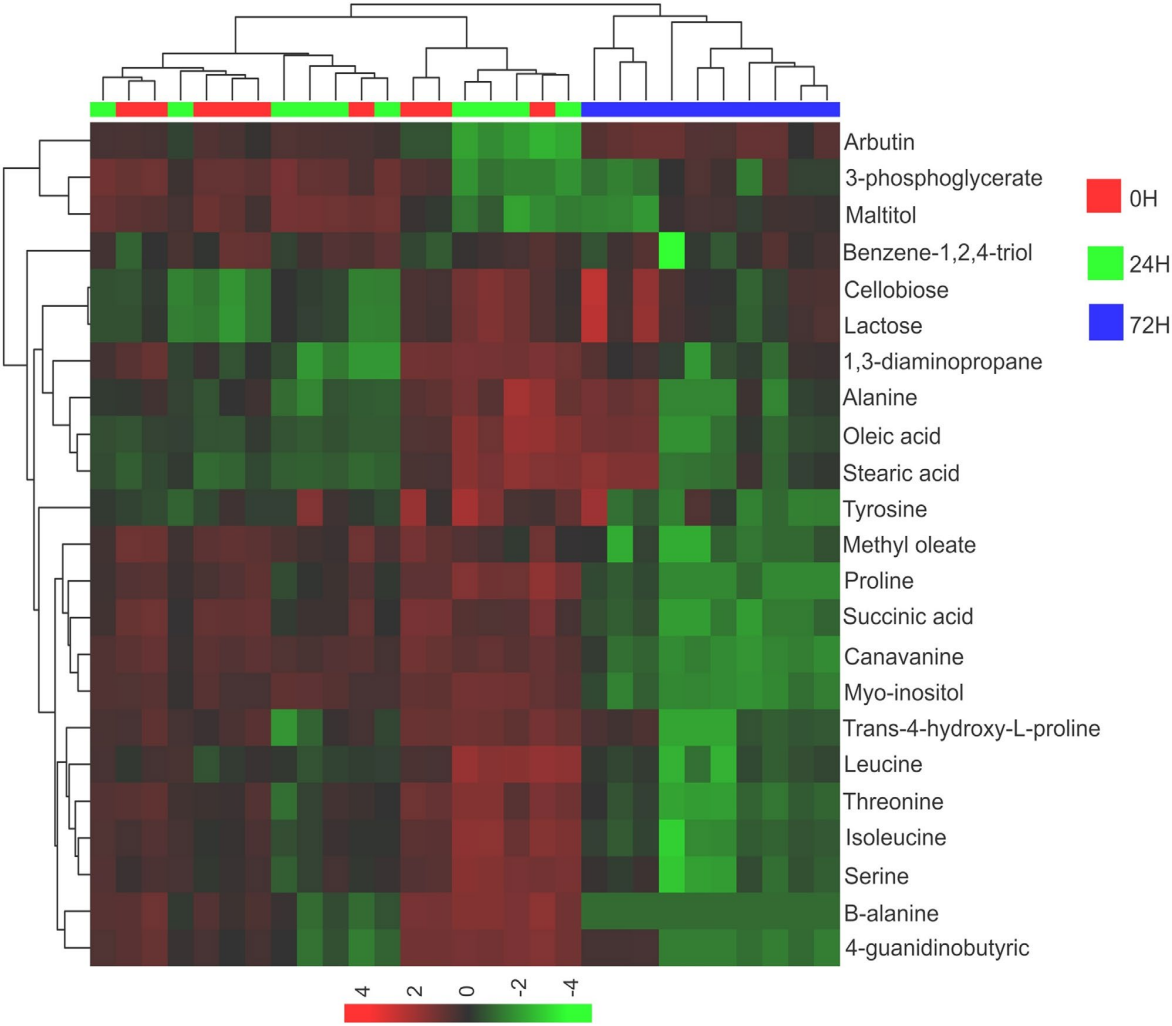
Heatmap and hierarchical clustering analysis of metabolites built with samples of *Acanthamoeba* (ATCC 30010) at the distinct times of encystation. The heatmap construction with the identified metabolites used the following parameters: Euclidean distance and average linkage. The *x* axis presents the evaluated time points, while the *y* axis presents the identified metabolites. The abundance of each metabolite is represented by the color scale, varying from −4 (light green) to +4 (light red).

Figure 4 provides a semi‐quantitative representation of the metabolic profile during encystation at 0, 24, and 72 h. Overall, metabolite abundance was higher in early phases (0 and 24 h) compared to the 72 h phase. However, some metabolites such as arbutin, cellobiose, and lactose showed increased levels at 72 h, coinciding with cyst predominance.

To identify metabolites driving group discrimination in the PLS‐DA models, we selected those with a variable importance in projection (VIP) score > 1.0 and/or statistical significance (*p* < 0.05, Student's *t*‐test or Mann–Whitney *U* test). Eighteen metabolites showed significant abundance differences across comparisons, with fold changes ranging from 0.19 to 2.22 (Table 1). Notably, arbutin, cellobiose, and lactose were markedly elevated in 72 h cyst samples compared to 0 h trophozoite samples (FC > 1.0, *p* < 0.05), consistent with their increased abundance in the heatmap (Figure 4).

**TABLE 1:** Stage‐specific metabolic variations during *Acanthamoeba* (ATCC 30010) encystation in Neff's saline solution.

Metabolites	24 × 0 h			72 × 0 h			72 × 24 h		
	VIP	*p*	FC^a^	VIP	*p*	FC^a^	VIP	*p*	FC^a^
1,3‐Diaminopropane	1.05		0.83						
3‐Phosphoglycerate					1.60E‐02	0.80			
4‐Guanidinobutiric acid				1.83	1.14E‐02	0.33		8.50E‐03	0.37
Arbutine					2.20E‐03	1.15	1.14	1.01E‐03	1.25
β‐Alanine				1.98	1.26E‐03	−∞	1.94	7.69E‐04	−∞
Canavanine	1.02	4.89E‐03	0.95	1.61	2.80E‐04	0.60	1.63	1.83E‐04	0.63
Cellobiose					2.74E‐02	1.03			
Isoleucine				1.22	1.51E‐04	0.71	1.53	2.57E‐04	0.69
Lactose					2.74E‐02	1.03			
Leucine				1.03	3.75E‐03	0.62	1.4	2.48E‐03	0.55
Methyl oleate	1.67	1.00E‐06	0.83	1.4	2.80E‐04	0.41	1.22	2.46E‐04	0.50
Myo‐inositol				1.31	2.80E‐04	0.76	1.47	7.65E‐10	0.75
Proline				2.21	2.80E‐04	0.19	2.16	3.30E‐04	0.21
Serine				1.03	1.63E‐03	0.79	1.24	1.15E‐03	0.76
Succinic acid	2.64	1.00E‐06	0.77	2.22	2.80E‐04	0.29	1.92	3.20E‐05	0.37
Threonine				1.18	1.20E‐05	0.79	1.14	3.12E‐04	0.81
Tyrosine							1.06	3.12E‐02	0.76
trans‐4‐Hydroxy‐L‐proline	1.62		0.84	1.86	3.75E‐03	0.50	1.40	4.13E‐02	0.59

Abbreviation: VIP, variable importance in projection.

^a^ FC, Fold change calculated by the metabolite intensity ratio of the samples with a longer time of cultivation than those with a shorter time of cultivation. FC, −∞ means the metabolite was not detected at 72 h.

### 3.4 Pathway Analysis

To identify metabolic pathways potentially involved in encystation, we performed pathway enrichment analysis using MBrole 2.0 with KEGG data from related organisms *Entamoeba histolytica* , *Entamoeba dispar*, and *Dictyostelium discoideum*. Significant metabolic pathways are listed in Table 2. Only the comparisons between 72 h versus 0 h and 72 h versus 24 h exhibited statistically significant differentially regulated pathways. In the 72 h versus 0 h comparison, glycine/serine/threonine metabolism and aminoacyl‐tRNA biosynthesis pathways were differentially regulated. The latter pathway was enriched in comparison between 24 h and 72 h.

**TABLE 2:** Metabolic pathways enriched from differential metabolites identified in samples of *Acanthamoeba* (ATCC 30010) induced to encyst at 0, 24, and 72 h.

Encystation times	Metabolic pathways^a^	Metabolites associated with the pathway	Organism used in enrichment
72 × 0 h	Glycine, serine, and threonine metabolism	3‐phosphoglycerate, threonine and serine	*Entamoeba histolytica, Entamoeba dispar Dictyostelium discoideum*
	Aminoacyl‐tRNA biosynthesis	β‐alanine, serine and threonine	*Entamoeba histolytica, Entamoeba dispar Dictyostelium discoideum*
72 × 24 h	Aminoacyl‐tRNA biosynthesis	β‐alanine, serine, threonine and tyrosine	*Entamoeba histolytica, Entamoeba díspar and Dictyostelium discoideum*

^a^ A *p* < 0.05 for every enrichment demonstrates the significance of pathway analysis.

## 4 Discussion

In this study, we investigated metabolic changes during *Acanthamoeba* encystation using an untargeted metabolomics approach. This strategy enables relative quantification of metabolites, providing a foundation for future targeted studies (Schrimpe‐Rutledge et al. 2016). By employing a nutrient deprivation medium to induce encystation (Neff et al. 1964), we analyzed three key stages of differentiation: the initial trophozoite‐dominated phase (0 h), followed by pre‐cyst (24 h) and mature cyst (72 h) stages. The progressive decrease in amino acid abundance as encystation progresses aligns with the overall metabolic downregulation expected during cyst formation. Bernard et al. (2022) demonstrated through multi‐omics analysis that suppression of ribosome biogenesis and protein synthesis pathways occurs within the first 8 h of encystation, marking early metabolic reorganization. Complementary work by de Obeso Fernández Del Valle et al. (2022) revealed post‐transcriptional regulation via increased intron retention events in encysting stages (24–48 h), particularly affecting genes involved in protein turnover and lipid metabolism. Our metabolomic profiling complements these findings by revealing how these molecular events manifest in functional metabolic changes during cyst formation.

The depletion of amino acids may also reflect their active catabolism to meet energy demands. In *Entamoeba invadens* , amino acids are degraded to generate ATP during encystation (Jeelani et al. 2012). The parallels between *Entamoeba* and *Acanthamoeba* encystation, such as dormancy induced by nutrient stress and cyst wall synthesis, suggest amino acids in *Acanthamoeba* may serve as a carbon source for biosynthetic pathways.

Among the amino acids detected, canavanine showed higher levels in trophozoites but was downregulated in cysts. Not previously reported in *Acanthamoeba*, canavanine is structurally analogous to L‐arginine and can be mis‐incorporated into proteins, leading to non‐functional polypeptides (Rodgers and Shiozawa 2008). While toxic to most organisms (Kamo et al. 2015), certain species of plants and the beetle *Caryedes brasiliensis* repurpose canavanine as a nitrogen storage molecule for amino acid synthesis (Rosenthal et al. 1982). Its biological effects appear context‐dependent, having been associated with growth inhibition in nitrogen‐fixing algae (Kumar and Kumar 1981). Benedetto et al. (2003) showed that exogenous L‐canavanine blocks nitric oxide (NO) production in microglial cells by inhibiting the enzyme iNOS (inducible nitric oxide synthase). As a result, it disrupts the immune system's ability to control *Acanthamoeba* growth through cytokine‐mediated defenses. Our discovery of endogenous canavanine in trophozoites and its stage‐specific downregulation in cysts suggests this non‐proteinogenic amino acid may function as an intrinsic immunomodulator. This raises the intriguing possibility that endogenous canavanine in *Acanthamoeba* trophozoites may contribute to immune evasion, a hypothesis warranting future investigation.

In addition to novel metabolites like canavanine, our metabolomic analysis detected several components previously reported in *Acanthamoeba*. For example, we identified hydroxyproline, an amino acid derived from proline hydroxylation first described by Bauer (1967) in the protozoa. We also observed 1,3‐diaminopropane (DAP) as the most abundant polyamine in trophozoites, with levels decreasing significantly in cysts. This finding aligns with earlier studies reporting high DAP concentrations in *Acanthamoeba* active‐stage and marked reduction during encystation (Poulin et al. 1984; Kim et al. 1987; Zhu et al. 1989). Considering the role of polyamines in growth, cell differentiation, and nucleic acid/protein synthesis across diverse organisms (Gevrekci 2017; Handa et al. 2018), their metabolic pathways represent possible chemotherapeutic targets against *Acanthamoeba* infections.

The primary structural component of the cyst wall, cellulose (Dudley et al. 2009; Lloyd 2014), is synthesized through uridine diphosphate (UDP) glucose polymerization, with glycogen metabolism potentially supplying precursor molecules (Stewart and Weisman 1974; Potter and Weisman 1976). We detected cellobiose, a disaccharide subunit and potential metabolic byproduct of cellulose, across all encystation stages, with significant accumulation in mature cysts (72 h). Calcofluor white staining studies have demonstrated that polymeric cellulose deposition begins during early encystation and progressively intensifies through later stages of cyst maturation (Lorenzo‐Morales et al. 2008). Our metabolomic data complement these findings by indicating late‐stage cellobiose accumulation, suggesting dynamic cellulose biosynthesis and remodeling during wall assembly. Of note, complete wall assembly determines environmental resistance, and thus, specifically targeting cellulose biosynthesis could be a strategy to impair cyst formation and viability (Anwar et al. 2018; Garajová et al. 2019; Lakhundi et al. 2015).

Before the 2020s, no studies have reported lactose in *Acanthamoeba*'s biochemical composition. However, Dudley et al. (2009) analyzed trophozoites, cysts, and cyst walls, identifying high percentages of galactose and glucose along with trace amounts of mannose and xylose. Their study focused on carbohydrate profiling using GC–MS‐based glycosyl composition analysis, which included enzymatic pre‐treatment with amyloglucosidase, papain, DNase, RNase, and Proteinase K. In contrast, our methodology omitted enzymatic treatment to enable rapid metabolite extraction and metabolic quenching, thereby preventing metabolite degradation or unintended biochemical reactions. This key methodological difference suggests that the enzymatic treatments used by Dudley et al. (2009) hydrolyzed glycosidic bonds in lactose, explaining its absence in their carbohydrate profile. Simau et al. (2024) recently investigated lactase‐mediated hydrolysis of cyst wall carbohydrates. These authors demonstrated that exogenous lactase treatment disrupts *Acanthamoeba* cysts by cleaving lactose into glucose and galactose, indirectly supporting our findings that intact lactose also exists in cysts under non‐enzymatic conditions. Their findings, combined with our detection of lactose, suggest that this disaccharide could serve as both a structural component and an energy reserve.

In cystic stages, we detected increased levels of arbutin, a glycoside derived from hydroquinone, commonly found in food and medicinal plants. It acts as a depigmenting agent by inhibiting tyrosinase (Funayama et al. 1995; Zhou et al. 2019) and exhibits antimicrobial, antioxidant, and anti‐inflammatory properties, making it valuable in cosmetics and medicine (Xu et al. 2015; Zhou et al. 2019). The detection of arbutin in *Acanthamoeba* is a notable finding, as this compound is typically associated with plant organisms. Previous research has shown that *Acanthamoeba* trophozoites increase antioxidant activity in response to oxidative stress (Motavalli et al. 2018). Considering arbutin's antioxidant properties and the resilience of cysts, its presence in late‐stage encystation may suggest a protective mechanism against external oxidative stress. Further studies could elucidate arbutin's origin and functional role in *Acanthamoeba* cysts, including whether it is synthesized endogenously or acquired from the environment.

Given the limited annotations of *Acanthamoeba* metabolic pathways in *KEGG* (only glycolysis, TCA cycle, and starch metabolism), we mapped metabolites to pathways using comparative data from *E. histolytica* , *E. dispar* , and *D. discoideum*, as these organisms share conserved metabolic adaptations for surviving environmental stress through dormant stages. (Schaap and Schilde 2018; Samba‐Louaka et al. 2023). One metabolic pathway enriched was glycine, serine, and threonine metabolism, in which the metabolites are decreased in the cyst stages. In the targeted study by Tsai et al. (2023), this same pathway was implicated in amino acid homeostasis achievement after long‐term exposure of *Acanthamoeba* to the coculture media. Another recent study on *Trypanosoma cruzi* demonstrated that serine and threonine act as critical carbon sources during nutritional stress, sustaining mitochondrial membrane potential (ΔΨm) and ATP production via oxidative phosphorylation (Alencar et al. 2025). Regulation of this pathway in *Acanthamoeba* may represent a strategy to repurpose these amino acids for energy maintenance and stress resilience during dormancy.

Another highlighted metabolic pathway, the aminoacyl‐tRNA biosynthesis, includes components involved in the essential first step of protein synthesis, where amino acids are activated and covalently attached to their cognate tRNAs by aminoacyl‐tRNA synthetases (Rubio Gomez and Ibba 2020). The enrichment of this pathway suggests *Acanthamoeba* regulates a ready pool of amino acids and charged tRNAs that could enable a rapid resumption of protein synthesis during excystation.

This study has two considerations for interpretation. First, the partial asynchrony in encystation resulted in mixed populations of trophozoites, pre‐cysts, and mature cysts during intermediate phases. Although the cyst stage (≥ 85% purity at 72 h) exhibited evident metabolic downregulation and PCA analysis reinforced metabolic distinctions, the previous time point (24 h) contained overlapping stages. This heterogeneity likely contributed to the lack of distinct clustering in trophozoite and pre‐cyst‐predominant samples and could complicate the identification of phase‐specific metabolic signatures. Second, our data derived from a long‐term cultured reference strain, which, coupled with the documented diversity in *Acanthamoeba* genus, could impact the generalizability of the metabolic findings to other isolates. Future comparative metabolomics across diverse species and strains may differentiate conserved encystation pathways from strain‐specific adaptations.

In conclusion, the untargeted approach used in this study identified metabolites not previously reported in *Acanthamoeba* and confirmed a general downregulation of metabolic activity in cysts, consistent with an energy‐conserving dormant state. Future investigations employing targeted assays and larger sampling can help to broaden the knowledge on cell differentiation in this protozoan.

## Supporting information


**Figure S1:** Principal component analysis (PCA) and partial least squares‐discriminant analysis (PLS‐DA) of metabolite identified from *Acanthamoeba* (ATCC 30010) samples at different times throughout in vitro encystation. A. PCA: Quality parameters of the model: *R* ^2^ = 0.676 *Q* ^2^ = 0.578. B. PSL‐DA: Quality parameters of the model: *R* ^2^ = 0.607 *Q* ^2^ = 0.469. Analyses were performed in cultures submitted to encystation at every 24 h for up to 72 h. The model was built with total features (Pareto scaled) detected by GC–MS in the presence of Quality Control (QCs). The clustering of the quality controls proved to be satisfactory, demonstrating the stability and reproducibility of the technique. Since it is an unsupervised method, the trend of clustering of the samples according to the culture time is relevant to the analysis.
**Table S1:** GC–MS‐detected metabolites and their physicochemical properties during in vitro encystation of *Acanthamoeba* (ATCC 30010).

## Data Availability

The data that supports the findings of this study are available in the Supporting Information—S1 of this article.
