# Supplementary material for: Metabolic Profile Associated With Encystation in Acanthamoeba
Source: J Eukaryot Microbiol. 2025 Jul 30;72(5):e70034. doi: 10.1111/jeu.70034 (PMC12310340; doi:10.1111/jeu.70034)
Supplement: Supplementary file 1 — Figure S1: Principal component analysis (PCA) and partial least squares‐discriminant analysis (PLS‐DA) of metabolite identified from Acanthamoeba (ATCC 30010) samples at different times throughout in vitro encystation. A. PCA: Quality parameters of the model: R 2 = 0.676 Q 2 = 0.578. B. PSL‐DA: Quality parameters of the model: R 2 = 0.607 Q 2 = 0.469. Analyses were performed in cultures submitted to encystation at every 24 h for up to 72 h. The model was built with total features (Pareto scaled) detected by GC–MS in the presence of Quality Control (QCs). The clustering of the quality controls proved to be satisfactory, demonstrating the stability and reproducibility of the technique. Since it is an unsupervised method, the trend of clustering of the samples according to the culture time is relevant to the analysis. Table S1: GC–MS‐detected metabolites and their physicochemical properties during in vitro encystation of Acanthamoeba (ATCC 30010). [file JEU-72-e70034-s001.docx]

**SUPPLEMENTARY MATERIAL**


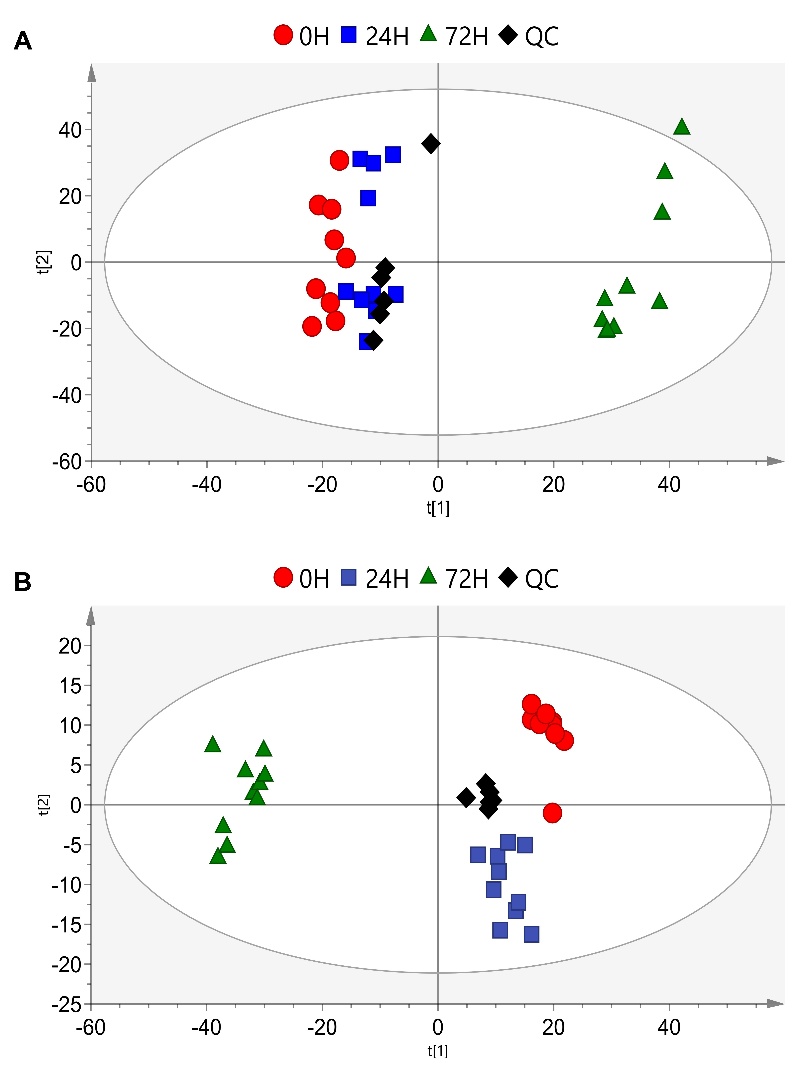


**Figure S1.** Principal component analysis (PCA) and partial least squares-discriminant analysis (PLS-DA) of metabolite identified from *Acanthamoeba* (ATCC 30010) samples at different times throughout *in vitro* encystation. A. PCA: Quality parameters of the model: R^2^= 0.676 Q^2^ = 0.578. B. PSL-DA: Quality parameters of the model: R^2^= 0.607 Q^2^ = 0.469. Analyses were performed in cultures submitted to encystation at every 24 hours for up to 72 hours. The model was built with total features (Pareto scaled) detected by GC-MS in the presence of Quality Control (QCs). The clustering of the quality controls proved to be satisfactory, demonstrating the stability and reproducibility of the technique. Since it is an unsupervised method, the trend of clustering of the samples according to the culture time is relevant to the analysis.

**Table S1.** GC-MS-detected metabolites and their physicochemical properties during in vitro encystation of *Acanthamoeba* (ATCC 3010)

| **Metabolite** | **Molecular Formula** | **Monoisotopic mass (g/mol)** | **Biochemical classification** |
| --- | --- | --- | --- |
| Myo-inositol | C_6_H_12_O_6_ | 180.063 | Alcohols and polyols (4.35%) |
| Alanine | C_3_H_7_NO_2_ | 89.048 | Amino acids, peptides and conjugates  (47.83%) |
| Leucine | C_6_H_13_NO_2_ | 131.095 |  |
| Proline | C_5_H_9_NO_2_ | 115.063 |  |
| Isoleucine | C_6_H_13_NO_2_ | 131.095 |  |
| Serine | C_3_H_7_NO_3_ | 105.042 |  |
| Threonine | C_4_H_9_NO_3_ | 119.058 |  |
| β-Alanine | C_3_H_7_NO_2_ | 89.048 |  |
| trans-4-Hydroxy-L-proline | C_5_H_9_NO_3_ | 131.058 |  |
| Tyrosine | C_9_H_11_NO_3_ | 181.074 |  |
| Canavanine | C_5_H_12_N_4_O_3_ | 176.091 |  |
| 4-Guanidinobutyric acid | C_5_H_11_N_3_O_2_ | 145.085 |  |
| 3-Phosphoglycerate | C_3_H_7_O_7_P | 186.056 | Carbohydrates (26.09%) |
| Arbutin | C_12_H_16_O_7_ | 272.090 |  |
| Cellobiose | C_12_H_22_O_11_ | 342.116 |  |
| Lactose | C_12_H_22_O_11_ | 342.116 |  |
| Maltitol | C_12_H_24_O_11_ | 344.132 |  |
| Succinic acid | C_4_H_6_O_4_ | 118.027 |  |
| Methyl oleate | C_19_H_36_O_2_ | 296.271 | Fatty acids and conjugates  (13.04%) |
| Oleic acid | C_18_H_34_O_2_ | 282.256 |  |
| Stearic acid | C_18_H_36_O_2_ | 284.271 |  |
| 1,3-Diaminopropane | C_3_H_10_N_2_ | 74.084 | Organic nitrogen compounds (4.35%) |
| Benzene-1,2,4-triol | C_6_H_6_O_3_ | 126.032 | Phenols and derivatives (4.35%) |
